# Supplementary material for: Molecular Prevalence, Genetic Diversity, and Tissue Tropism of Bartonella Species in Small Mammals from Yunnan Province, China
Source: Animals (Basel). 2024 Apr 28;14(9):1320. doi: 10.3390/ani14091320 (PMC11083988; doi:10.3390/ani14091320)
Supplement: Supplementary file 1 [file animals-14-01320-s001.zip › animals-2962978-supplementary figures.pdf]

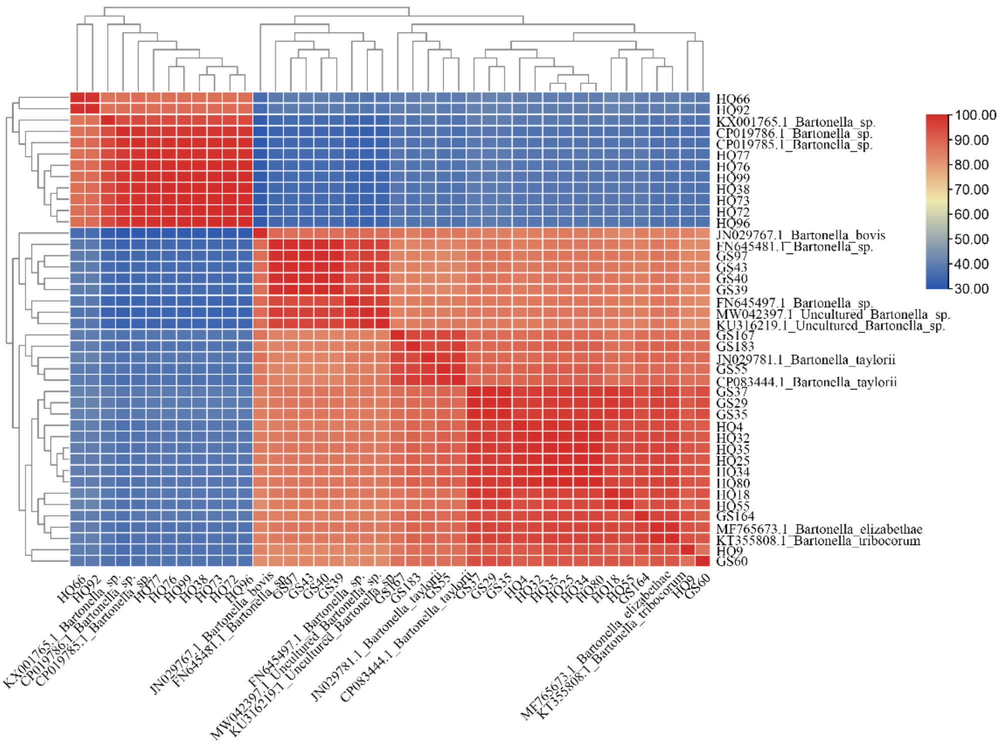

Fig. S1. Identity comparisons of nucleotide sequence of *Bartonella ssrA* gene (301 bp) in small mammals from this study.

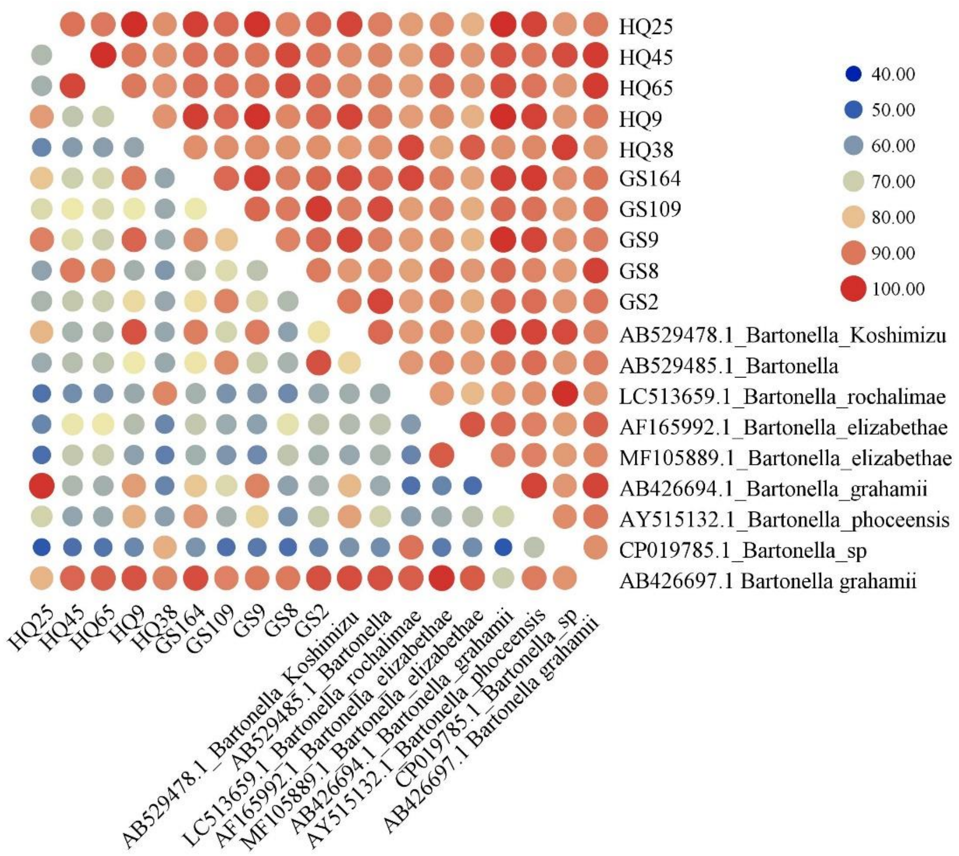

Fig. S2. Identity comparisons of nucleotide and amino acid sequence of *Bartonella rpoB* gene

(866bp) in small mammals from this study.

Note: Top right indicates nucleotide sequence identity, while bottom left shows amino acid sequence homology.

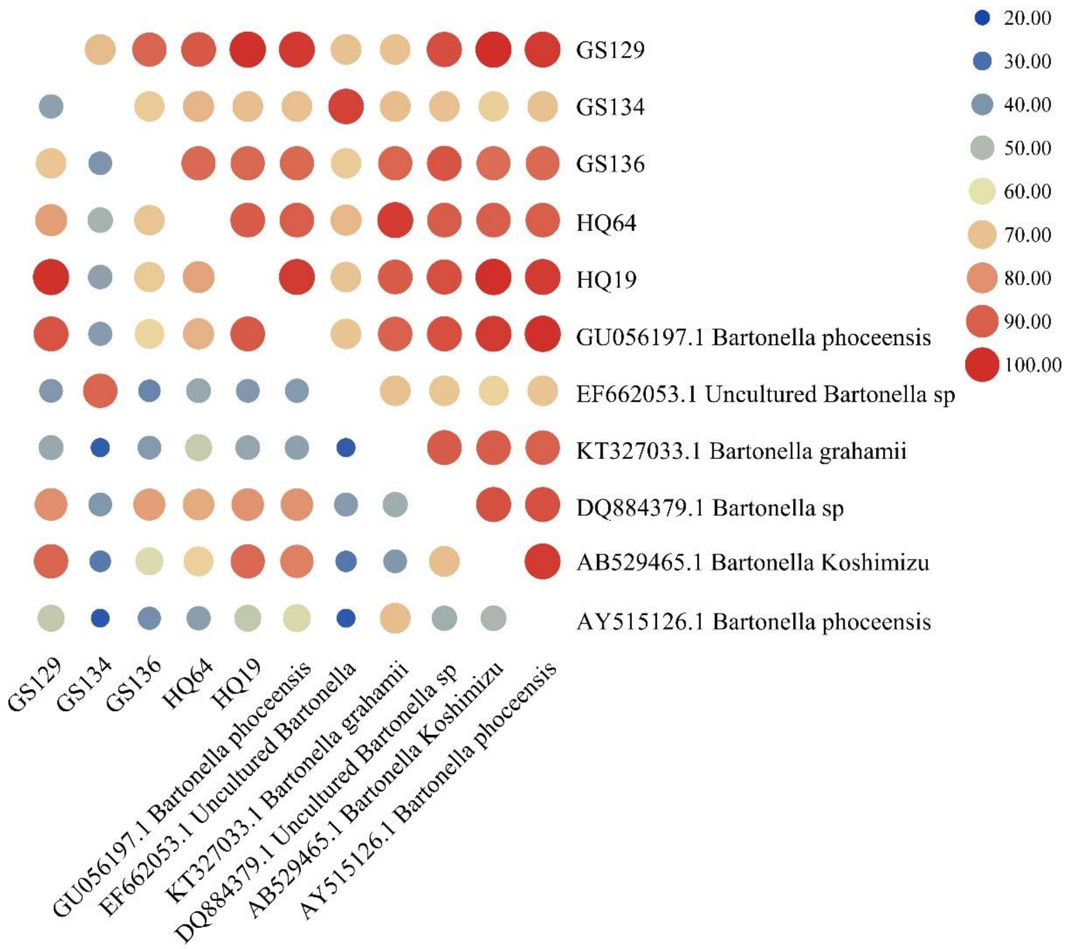

Fig. S3. Identity comparisons of nucleotide and amino acid sequence of *Bartonella gltA* gene (375bp) in small mammals from this study.

Note: Top right indicates nucleotide sequence identity, while bottom left shows amino acid sequence homology.

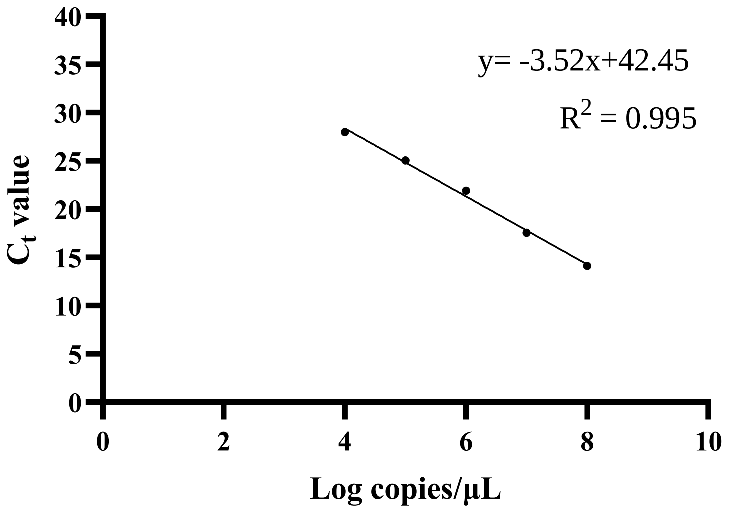

Fig. S4. Standard curve. The equation of the standard curve is y = -3.477x + 42.23, with a correlation coefficient (R^2) of 0.995. The slope is -3.477, and the amplification efficiency (E%) is 93.9%.

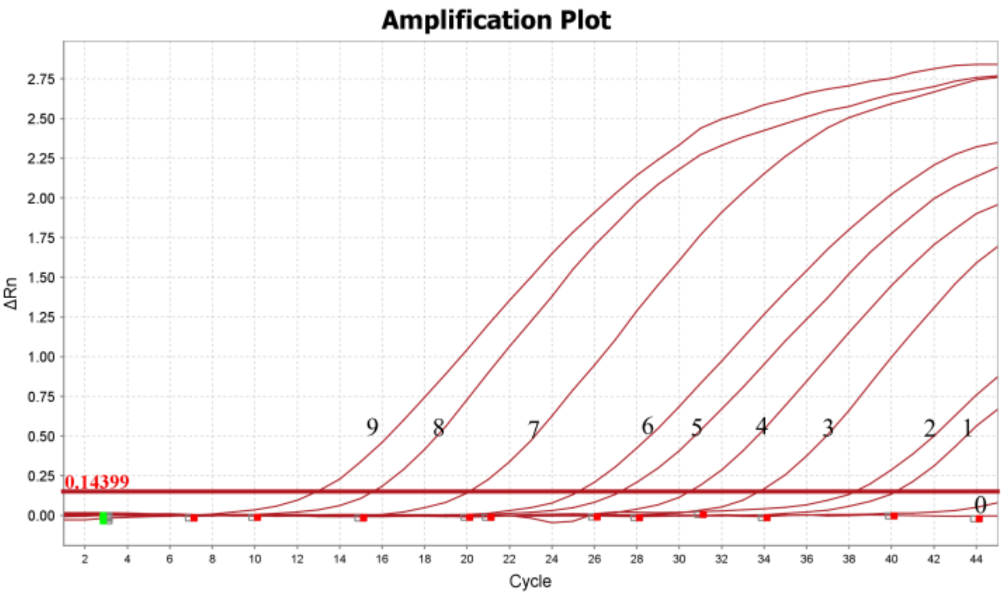

Fig. S5. Sensitivity test results. Copies ranged from 1.0×10^{9} to 1.0×10^{1} copies/L; 0 indicates the negative control.

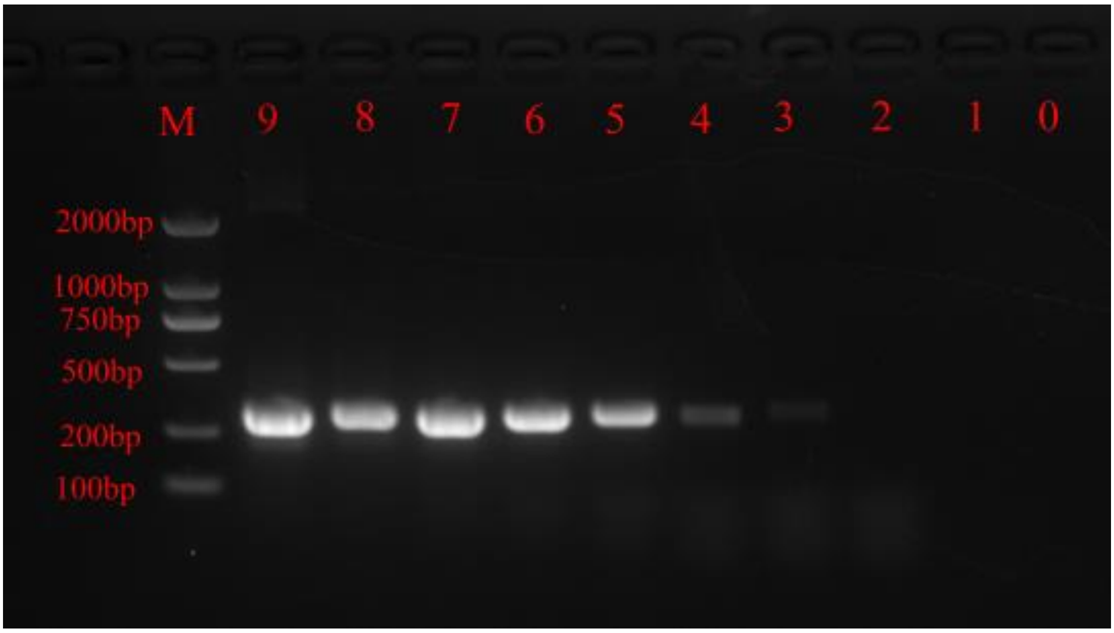

Fig. S6. Conventional PCR results. M: Trans2K DNA Marker; 9-1:1.0×10^{9}-1.0×10^{1}; 0: negative control.
