# Supplementary material for: Molecular Prevalence, Genetic Diversity, and Tissue Tropism of Bartonella Species in Small Mammals from Yunnan Province, China
Source: Animals (Basel). 2024 Apr 28;14(9):1320. doi: 10.3390/ani14091320 (PMC11083988; doi:10.3390/ani14091320)
Supplement: Supplementary file 1 [file animals-14-01320-s001.zip › animals-2962978-supplementary tables.pdf]

Table S1 Identity comparisons of nucleotide sequence of *Bartonella ssrA* gene (301 bp) from small mammals in this study

	HQ35	HQ4	HQ9	HQ18	HQ25	HQ32	HQ34	HQ38	HQ55	HQ66	HQ73	HQ72	HQ77	HQ80	HQ92	HQ96	HQ76	HQ99	GS183	GS97	GS43	GS60	GS164	GS40	GS39	GS29	GS35	GS37	GS55	GS167	1	2	3	4	5	6	7	8	9	10	11	12
**HQ35**	100.0																																								
**HQ4**	98.9	100.0																																							
**HQ9**	93.1	92.1	100.0																																						
**HQ18**	96.2	95.2	91.5	100.0																																					
**HQ25**	100.0	98.9	93.1	96.2	100.0																																				
**HQ32**	99.5	98.4	92.6	95.7	99.5	100.0																																			
**HQ34**	100.0	98.9	93.1	96.2	100.0	99.5	100.0																																		
**HQ38**	38.2	38.2	37.1	38.2	38.2	38.2	38.2	100.0																																	
**HQ55**	95.2	94.1	90.5	97.9	95.2	94.6	95.2	38.2	100.0																																
**HQ66**	39.8	40.3	39.7	41.4	39.8	39.8	39.8	88.3	41.4	100.0																															
**HQ73**	38.2	38.2	37.1	38.2	38.2	38.2	38.2	100.0	38.2	88.3	100.0																														
**HQ72**	38.2	38.2	37.1	38.2	38.2	38.2	38.2	100.0	38.2	88.3	100.0	100.0																													
**HQ77**	38.2	38.2	37.1	38.2	38.2	38.2	38.2	99.5	38.2	87.8	99.5	99.5	100.0																												
**HQ80**	100.0	98.9	93.1	96.2	100.0	99.5	100.0	38.2	95.2	39.8	38.2	38.2	38.2	100.0																											
**HQ92**	39.8	40.3	39.7	41.4	39.8	39.8	39.8	88.8	41.4	99.5	88.8	88.8	88.3	39.8	100.0																										
**HQ96**	38.2	38.2	37.1	38.2	38.2	38.2	38.2	100.0	38.2	88.3	100.0	100.0	99.5	38.2	88.8	100.0																									
**HQ76**	38.2	38.2	37.1	38.2	38.2	38.2	38.2	100.0	38.2	88.3	100.0	100.0	99.5	38.2	88.8	100.0	100.0																								
**HQ99**	38.2	38.2	37.1	38.2	38.2	38.2	38.2	100.0	38.2	88.3	100.0	100.0	99.5	38.2	88.8	100.0	100.0	100.0																							
**GS183**	90.9	89.8	88.4	88.2	90.9	90.3	90.9	37.7	87.6	39.8	37.7	37.7	37.7	90.9	39.8	37.7	37.7	37.7	100.0																						
**GS97**	86.8	85.7	83.9	86.8	86.8	86.2	86.8	35.4	85.7	39.6	35.4	35.4	35.4	86.8	39.6	35.4	35.4	35.4	84.7	100.0																					
**GS43**	86.8	85.7	83.9	86.8	86.8	86.2	86.8	35.4	85.7	39.6	35.4	35.4	35.4	86.8	39.6	35.4	35.4	35.4	84.7	100.0	100.0																				
**GS60**	92.5	91.4	87.8	93.0	92.5	91.9	92.5	37.6	92.5	39.2	37.6	37.6	37.6	92.5	39.2	37.6	37.6	37.6	88.2	83.6	83.6	100.0																			
**GS164**	95.7	94.6	94.2	94.1	95.7	95.2	95.7	38.7	93.0	39.8	38.7	38.7	38.7	95.7	39.8	38.7	38.7	38.7	89.8	85.2	85.2	90.3	100.0																		
**GS40**	86.8	85.7	83.9	86.8	86.8	86.2	86.8	35.4	85.7	39.6	35.4	35.4	35.4	86.8	39.6	35.4	35.4	35.4	84.7	100.0	100.0	83.6	85.2	100.0																	
**GS39**	86.8	85.7	83.9	86.8	86.8	86.2	86.8	35.4	85.7	39.6	35.4	35.4	35.4	86.8	39.6	35.4	35.4	35.4	84.7	100.0	100.0	83.6	85.2	100.0	100.0																
**GS29**	97.9	96.8	92.1	98.4	97.9	97.3	97.9	39.3	97.3	40.8	39.3	39.3	39.3	97.9	40.8	39.3	39.3	39.3	89.3	86.8	86.8	94.1	94.6	86.8	86.8	100.0															
**GS35**	97.9	96.8	92.1	98.4	97.9	97.3	97.9	39.3	97.3	40.8	39.3	39.3	39.3	97.9	40.8	39.3	39.3	39.3	89.3	86.8	86.8	94.1	94.6	86.8	86.8	100.0	100.0														
**GS37**	97.9	96.8	92.1	98.4	97.9	97.3	97.9	39.3	97.3	40.8	39.3	39.3	39.3	97.9	40.8	39.3	39.3	39.3	89.3	86.8	86.8	94.1	94.6	86.8	86.8	100.0	100.0	100.0													
**GS55**	91.4	90.3	87.8	88.2	91.4	90.9	91.4	37.7	87.6	39.3	37.7	37.7	37.7	91.4	39.3	37.7	37.7	37.7	95.7	83.6	83.6	88.2	89.3	83.6	83.6	89.8	89.8	89.8	100.0												
**GS167**	89.3	88.2	86.8	87.1	89.3	88.7	89.3	38.2	86.6	39.8	38.2	38.2	38.2	89.3	39.8	38.2	38.2	38.2	95.2	84.1	84.1	87.6	88.2	84.1	84.1	88.7	88.7	88.7	95.2	100.0											
**1**	37.7	37.7	36.6	37.7	37.7	37.7	37.7	99.5	37.7	88.8	99.5	99.5	98.9	37.7	89.4	99.5	99.5	99.5	37.2	34.9	34.9	37.0	38.2	34.9	34.9	38.7	38.7	38.7	37.2	37.7	100.0										
**2**	86.8	85.7	83.9	86.2	86.8	86.2	86.8	33.9	85.2	38.0	33.9	33.9	33.9	86.8	38.0	33.9	33.9	33.9	85.2	96.8	96.8	83.6	85.2	96.8	96.8	86.2	86.2	86.2	85.2	84.7	33.3	100.0									
**3**	86.2	85.2	83.3	85.7	86.2	85.7	86.2	34.4	84.7	38.5	34.4	34.4	34.4	86.2	38.5	34.4	34.4	34.4	85.2	95.8	95.8	83.1	84.7	95.8	95.8	85.7	85.7	85.7	85.7	84.7	33.9	98.9	100.0								
**4**	38.7	38.7	37.6	38.7	38.7	38.7	38.7	94.7	38.7	87.2	94.7	94.7	94.2	38.7	87.8	94.7	94.7	94.7	37.7	35.4	35.4	37.6	38.2	35.4	35.4	39.8	39.8	39.8	37.7	38.2	95.2	33.9	34.4	100.0							
**5**	85.6	84.6	84.8	84.0	85.6	85.1	85.6	31.9	83.0	35.6	31.9	31.9	31.9	85.6	35.6	31.9	31.9	31.9	85.1	88.4	88.4	82.5	85.1	88.4	88.4	85.1	85.1	85.1	85.6	87.2	31.4	88.9	88.4	31.9	100.0						
**6**	86.2	85.2	83.3	86.2	86.2	85.7	86.2	34.9	85.2	39.1	34.9	34.9	34.9	86.2	39.1	34.9	34.9	34.9	84.1	99.5	99.5	83.1	84.7	99.5	99.5	86.2	86.2	86.2	84.1	84.7	34.4	97.4	96.3	34.9	88.9	100.0					
**7**	37.2	37.2	36.1	37.2	37.2	37.2	37.2	98.4	37.2	87.8	98.4	98.4	97.9	37.2	88.3	98.4	98.4	98.4	36.7	34.4	34.4	36.5	37.7	34.4	34.4	38.2	38.2	38.2	37.7	37.2	98.9	32.8	33.3	94.2	30.9	33.9	100.0				
**8**	86.7	85.6	83.8	86.2	86.7	86.2	86.7	34.6	85.1	38.7	34.6	34.6	34.6	86.7	38.7	34.6	34.6	34.6	85.1	94.7	94.7	83.0	85.1	94.7	94.7	86.2	86.2	86.2	85.1	84.6	34.0	97.9	96.8	34.6	88.8	95.2	33.5	100.0			
**9**	96.2	95.2	93.7	95.7	96.2	95.7	96.2	37.2	94.6	39.8	37.2	37.2	37.2	96.2	39.8	37.2	37.2	37.2	90.3	86.8	86.8	92.5	96.2	86.8	86.8	96.2	96.2	96.2	89.3	88.2	36.7	86.8	86.2	37.7	84.6	86.2	36.1	86.7	100.0		
**10**	96.2	95.2	93.7	95.7	96.2	95.7	96.2	37.7	94.6	40.3	37.7	37.7	37.7	96.2	40.3	37.7	37.7	37.7	90.3	86.2	86.2	92.5	96.2	86.2	86.2	96.2	96.2	96.2	89.3	88.2	37.2	86.2	85.7	38.2	84.6	85.7	36.1	86.2	99.5	100.0	
**11**	90.9	89.8	88.4	88.2	90.9	90.3	90.9	37.2	87.6	39.3	37.2	37.2	37.2	90.9	39.3	37.2	37.2	37.2	97.3	84.1	84.1	88.2	89.8	84.1	84.1	89.3	89.3	89.3	98.4	95.7	36.7	85.7	86.2	37.2	86.2	84.7	36.1	85.6	89.8	89.8	100.0	
**12**	89.8	88.7	88.4	88.7	89.8	89.3	89.8	38.2	88.2	40.8	38.2	38.2	38.2	89.8	40.8	38.2	38.2	38.2	95.2	84.1	84.1	88.7	89.8	84.1	84.1	89.3	89.3	89.3	97.3	95.7	37.7	85.7	86.2	38.2	87.8	84.7	37.2	85.6	89.8	89.8	97.9	100.0

1: CP019785.1 *Bartonella* sp. 2: MW042397.1 Uncultured *Bartonella* sp. 3: KU316219.1 Uncultured *Bartonella* sp. 4: KX001765.1 *Bartonella* sp.5: JN029767.1 *Bartonella* bovis.6:FN645481.1 *Bartonella* sp.;7: CP019786.1 *Bartonella* sp.8:FN645497.1 *Bartonella* sp.9: MF765673.1 .10: KT355808.1 *Bartonella* tribocorum.11: CP083444.1 *Bartonella* taylorii.12: JN029781.1 *Bartonella* taylorii

Table S2 Identity comparisons of nucleotide and amino acid sequence of *Bartonella rpoB* gene (866bp) in small mammals in this study

	HQ25	HQ45	HQ65	HQ9	HQ38	GS164	GS109	GS9	GS8	GS2	1	2	3	4	5	6	7	8	9
**HQ25**		90.6	89.9	99.9	86.7	97.6	91.9	99.3	88.6	91.9	96.9	89.2	84.8	87.2	82.2	100.0	97.1	85.9	89.6
**HQ45**	66.3		99.8	90.0	86.6	91.1	90.9	90.4	96.2	90.7	87.4	88.2	84.9	91.9	86.5	94.9	90.2	96.0	98.3
**HQ65**	64.7	96.7		89.9	86.4	91.1	90.5	90.0	95.9	90.7	87.4	88.2	84.9	91.9	86.5	95.5	90.2	86.0	98.3
**HQ9**	85.1	68.6	69.5		86.3	97.7	91.8	99.4	88.0	92.0	97.0	89.4	84.9	87.3	82.2	99.9	97.2	86.0	89.6
**HQ38**	57.1	60.9	61.4	62.6		86.9	87.0	87.1	86.2	86.9	85.4	86.9	96.4	84.2	93.7	86.7	87.7	97.6	86.6
**GS164**	79.3	70.2	71.2	89.9	62.8		92.0	97.6	89.2	92.1	95.9	90.5	96.4	89.1	84.1	97.6	98.3	86.8	90.4
**GS109**	72.0	74.6	72.0	74.3	63.6	74.3		92.1	89.7	98.1	89.2	95.8	85.0	87.8	82.8	91.9	90.8	85.7	90.4
**GS9**	88.8	72.6	70.0	92.6	63.9	87.3	79.6		88.3	91.9	96.9	89.2	85.4	87.0	81.9	99.3	97.0	86.4	89.3
**GS8**	62.3	89.6	87.7	64.6	60.2	66.6	72.2	67.9		89.5	85.6	87.1	84.3	91.0	85.6	92.0	88.1	85.5	97.5
**GS2**	65.7	68.9	69.8	76.7	63.2	76.1	88.3	71.9	66.6		89.6	96.9	85.1	88.0	83.1	91.9	91.3	85.9	90.5
**1**	81.5	65.2	66.1	95.1	62.9	89.0	70.9	89.3	61.9	75.6		91.9	85.5	87.2	84.4	96.9	97.0	96.1	88.4
**2**	63.8	67.2	68.1	74.6	63.3	75.2	87.3	69.9	65.2	95.3	77.4		85.6	88.0	85.5	89.2	91.3	86.0	89.3
**3**	53.8	58.5	59.3	63.3	87.9	64.9	59.5	60.5	57.9	63.2	64.3	63.9		85.4	81.0	85.4	87.1	99.7	86.5
**4**	57.1	73.9	74.9	66.8	57.4	68.8	63.9	61.9	73.2	68.2	66.4	68.2	60.7		94.6	92.0	88.8	85.2	93.3
**5**	53.2	68.9	69.8	61.8	55.9	64.5	59.9	57.9	68.2	64.5	63.1	65.7	57.0	93.9		89.0	88.8	85.2	87.9
**6**	99.1	66.1	64.6	84.9	57.0	79.1	71.8	88.5	62.1	65.5	81.2	63.6	53.6	57.0	53.0		97.1	85.9	97.1
**7**	70.8	62.5	63.4	82.7	61.5	85.5	64.6	77.3	58.9	69.5	84.3	70.8	61.4	63.9	67.6	70.6		87.2	90.0
**8**	49.2	53.5	54.2	57.6	82.5	59.6	53.5	55.2	53.2	57.0	59.5	58.4	90.6	55.2	58.4	49.1	67.7		86.7
**9**	81.7	92.3	93.2	95.0	88.5	95.7	88.6	89.6	89.6	95.3	95.7	95.3	93.5	99.3	93.8	69.8	89.5	86.2	

1: AB529478.1 *Bartonella Koshimizu;* 2: AB529485.1 *Bartonella sp* ;3: LC513659.1 *Bartonella rochalimae* ; 4: AF165992.1 *Bartonella elizabethae* ; 5: MF105889.1 *Bartonella elizabethae* ; 6:AB426694.1 *Bartonella grahamii* ; 7:AY515132.1 *Bartonella phoceensis* ; 8:CP019785.1 *Bartonella* sp; 9:AB426697.1 *Bartonella grahamii*

Table S3 Identity comparisons of nucleotide and amino acid sequence of *Bartonella gltA* gene (375bp) in small mammals in this study

	GS129	GS134	GS136	HQ64	HQ19	1	2	3	4	5	5
**GS129**		71.0	88.7	91.3	99.7	97.9	70.1	70.1	93.7	100.0	97.6
**GS134**	43.3		68.3	72.6	70.7	70.4	96.0	70.9	70.2	67.5	70.4
**GS136**	69.3	40.2		87.9	88.4	88.0	68.0	88.6	92.1	87.3	87.9
**HQ64**	77.2	48.0	69.3		91.0	90.4	72.0	97.4	90.5	90.2	90.2
**HQ19**	99.2	43.3	68.5	76.4		97.6	69.8	90.7	93.4	99.7	97.4
**1**	92.1	41.7	66.1	73.2	91.3		69.6	89.6	93.1	97.6	99.7
**2**	40.9	89.0	36.2	44.9	40.9	41.7		70.4	69.3	66.6	69.6
**3**	45.1	25.4	41.3	54.5	44.6	43.2	25.4		90.7	90.5	89.7
**4**	80.3	40.9	77.2	74.8	79.5	79.5	41.7	46.5		92.9	92.9
**5**	89.0	33.1	58.3	66.9	88.2	83.3	32.3	40.9	70.9		97.6
**6**	53.7	24.1	38.4	42.6	53.2	57.9	24.5	70.8	46.8	48.6	

1: GU056197.1 *Bartonella phoceensis*； 2： EF662053.1 Uncultured *Bartonella sp*;3: KT327033.1 *Bartonella grahamii* ; 4: DQ884379.1 *Bartonella sp*; 5: AB529465.1 *Bartonella Koshimizu*;6: AY515126.1 *Bartonella phoceensis*

Table S3. Identity comparisons of nucleotide and amino acid sequence of *Bartonella gltA* gene (375bp) in small mammals from this study.`

Table S4. The results of intra-group repeatability.

Number of copies/(copies/μL)	C_t values			Mean C_t	SD	CV (%)
	1	2	3			
10^8	14.2	14.3	14.3	14.3	0.08	0.5
10^7	17.6	17.5	17.4	17.5	0.06	0.5
10^6	21.6	22.3	21.7	21.9	0.4	1.8
10^5	24.8	25.1	25.1	25.0	0.2	0.8
10^4	27.6	27.8	28.3	27.9	0.3	1.3

C_t: Cycle Threshold; SD: Standard deviation; CV: Coefficient of variation.

Table 3. The results of inter-group repeatability

Number of copies/(copies/μL)	C_t values			Mean C_t	SD	CV (%)
	1	2	3			
10^8	14.8	14.3	14.3	14.5	0.2	1.8
10^7	19.1	18.7	18.8	18.8	0.1	1.0
10^6	22.8	22.5	22.3	22.5	0.2	1.1
10^5	25.7	25.1	26.1	25.6	0.4	0.8
10^4	29.4	28.3	28.8	28.9	0.5	1.9

C_t: Cycle Threshold; SD: Standard deviation; CV: Coefficient of variation.
